# The Future of Glioblastoma Therapy: Synergism of Standard of Care and Immunotherapy

**DOI:** 10.3390/cancers6041953

**Published:** 2014-09-29

**Authors:** Mira A. Patel, Jennifer E. Kim, Jacob Ruzevick, Gordon Li, Michael Lim

**Affiliations:** 1 Department of Neurosurgery, The Johns Hopkins University School of Medicine, 600 N. Wolfe St., Phipps Building Rm 123, Baltimore, MD 21287, USA; E-Mails: mpatel50@jhmi.edu (M.A.P.); jkim256@jhmi.edu (J.E.K.); jruzevick@jhmi.edu (J.R.); 2 Department of Neurosurgery, Stanford University Medical Center, 1201 Welch Rd., P309 MSLS, Stanford, CA 94305, USA; E-Mail: gordonli@stanford.edu

**Keywords:** glioblastoma, temozolomide, vaccine, immunotherapy

## Abstract

The current standard of care for glioblastoma (GBM) is maximal surgical resection with adjuvant radiotherapy and temozolomide (TMZ). As the 5-year survival with GBM remains at a dismal <10%, novel therapies are needed. Immunotherapies such as the dendritic cell (DC) vaccine, heat shock protein vaccines, and epidermal growth factor receptor (EGFRvIII) vaccines have shown encouraging results in clinical trials, and have demonstrated synergistic effects with conventional therapeutics resulting in ongoing phase III trials. Chemoradiation has been shown to have synergistic effects when used in combination with immunotherapy. Cytotoxic ionizing radiation is known to trigger pro-inflammatory signaling cascades and immune activation secondary to cell death, which can then be exploited by immunotherapies. The future of GBM therapeutics will involve finding the place for immunotherapy in the current treatment regimen with a focus on developing strategies. Here, we review current GBM therapy and the evidence for combination of immune checkpoint inhibitors, DC and peptide vaccines with the current standard of care.

## 1. Introduction

Glioblastoma (GBM) is the most prevalent type of primary brain tumor, with a median survival of approximately one year [1,2]. Poor median survival has provided an impetus for developing survival-prolonging or curative therapies [3,4,5]. Despite current treatment regimens, GBM continues to be associated with a <10% 5-year survival rate [3,6]. A number of recent advances have shown promise for combating resistance to chemotherapy, and combination radiotherapy and chemotherapy regimes have been successful in modestly improving survival [7].

The current standard of care for GBM is safe maximal surgical resection followed by radiotherapy and concurrent Temozolomide (TMZ), followed by adjuvant TMZ [7]. Retrospective analysis has demonstrated that the extent of glioblastoma resection including residual volume of tumor left in the tumor bed significantly impacts overall survival and recurrence [8,9,10]. While TMZ is the mainstay chemotherapy for GBM, carmustine (BCNU) wafers have shown promise in providing localized chemotherapy for high-grade glioma [7,11,12,13,14]. As a result of only modest improvements in survival emerging from the traditional triad of surgical excision, radiotherapy, and chemotherapy, there have been intensified research efforts studying immunotherapy as a more effective treatment paradigm [6,15,16]. Immunotherapy such as cancer vaccines have shown encouraging results in phase I and II clinical trials, and have even demonstrated synergistic effects with standard therapy [17,18,19,20,21]. Immunotherapy is a promising approach to complement and enhance the current chemoradiation regimen.

## 2. Temozolomide: The Current Chemotherapeutic Standard

TMZ is a second-generation DNA alkylating agent that induces thymine mispairing during DNA replication resulting in tumor cell G2/M phase arrest and autophagy [2,22,23]. In a correlative specimen analysis from EORTC 26981/NCIC CE.3, Hegi* et al.* found that those with a methylated MGMT promoter had markedly increased survival after receiving TMZ compared to those with unmethylated MGMT promoters, suggesting that stratification of individuals based on tumor MGMT methylation status may be a necessary part of future trials and treatment regimens [24,25]. TMZ is an oral medication that is capable of crossing the blood-brain barrier and accumulates in high concentrations in the brain and in highly angiogenic tumors such as GBM [25,26,27,28].

Initial studies in high grade gliomas showed that TMZ produced an objective response rate of 11% and an objective response of 47% [29]. Further phase II studies continued to support TMZ’s efficacy in providing moderate response rates (23.8%) in GBM with acceptable toxicities, even in those individuals with poor performance status and with relapsing GBM refractory to surgical and radiotherapy [30,31] (Table 1). Yung and colleagues demonstrated the superiority of TMZ over procarbazine (PCB) in a phase II trial that became the foundation for FDA approval of TMZ as a first-line chemotherapeutic agent for GBM; they found that the 6-month overall survival rate for 112 patients receiving TMZ was 60% compared to 44% for 113 PCB-treated patients (*p* = 0.019) and the median progression free survival (PFS) with TMZ therapy was significantly improved over PCB (12.4 weeks* vs.* 8.32 weeks, *p* = 0.0063) [32,33]. Later studies built upon these findings, confirming the moderate clinical response and acceptable safety profile of TMZ in various sub-populations, such as the elderly and those at first-relapse [34,35]. Robust population-based studies have shown that since the adoption of TMZ as a component of standard therapy for GBM, there has been modest but meaningful improvements in survival in the GBM patient population, with 2-year survival increasing from 7% in those cases diagnosed from 1993–1995 to 17% in 2005–2007 [36]. Importantly, in 2005 Stupp and colleagues definitively showed the superiority of adjunctive TMZ therapy when they found that radiotherapy plus TMZ resulted in a 2-year survival rate of 26.5%* vs.* 10.4% with radiotherapy alone [2].

**Table 1:** Phase II clinical trials measuring temozolomide efficacy in Glioblastoma.

Reference	Response	Study Population
Bower, *et al.* (1997) [29]	^1^ 11% (CI 6%–24%)	Progressive or recurrent high grade glioma
Brandes, *et al.* (2001) [30]	^2^ 22.5% (CI 9.5%–35%)	Recurrent high-grade glioma following surgery +RT and chemotherapy
Janinis, *et al.* (2000) [31]	^1^ 25% (CI 12%–36%)	Relapsing malignant glioma and poor performance status
Yung, *et al.* (2000) [32]	^3^ 60% (*p* = 0.019)	First relapse

Selected major studies reporting the clinical effect of temozolomide on glioblastoma. Response refers to major measure of response to therapy in each study. ^1^ Objective response rate. ^2^ Overall response rate. ^3^ 6-month overall survival rate. CI = 95% Confidence Interval.

Nevertheless, while the success of TMZ in clinical trials showed great promise for its overall efficacy, emerging TMZ resistance in the form of enhanced MGMT activity, acquired mismatch repair gene mutations, and the persistence of cancer stem cell sub-populations necessitated examination of resistance pathways and therapeutics for overcoming them [24,37,38,39,40].

### Managing Temozolomide Resistance

Hegi and colleagues and subsequent studies established that MGMT promoter methylation is an important prognostic indicator of response to TMZ therapy [24,37,41]. Patients with un-methylated MGMT experience decreased survival and responsiveness to TMZ therapy. *In vitro* studies introduced O^6^-benzylguanine as a candidate for overcoming TMZ resistance in tumors with unmethylated MGMT that is mediated by O^6^-alkylguanine DNA alkyltransferase (AGT), the protein encoded by gene MGMT [42,43,44]. An AGT substrate, O^6^-benzylguanine inactivates AGT and prohibits its DNA repair activity [44]. *In vitro* and *in vivo* studies demonstrated that O^6^-benzylguanine-mediated AGT inhibition enhances TMZ cytotoxicity, and in a Phase II clinical trial Quinn *et al.* established the safety and efficacy of O^6^-benzylguanine in restoring TMZ sensitivity in TMZ-resistant anaplastic glioma with a 16% response rate [42,43,44,45,46,47]. Other avenues for overcoming MGMT-mediated TMZ resistance in GBM involve dual inhibition of base excision repair and NAD+ biosynthesis; blocking base excision repair results in hyperactivation of poly(ADP-ribose) polymerase as the cell attempts to repair TMZ-mediated damage without the appropriate excision repair pathways, and the cell ultimately dies from energy depletion induced by the accumulation of repair intermediates [37]. Goellner and colleagues have found that TMZ-resistant GBM cells with elevated MGMT become sensitized to TMZ therapy after blocking both base excision repair and biosynthesis of the energy source, NAD+ [37]. Nevertheless, the most recent studies assessing the efficacy of O^6^-benzylguanine have not shown promising results, and the recent phase III RTOG 0525 trial failed to show that dose dense TMZ overcomes MGMT-mediated TMZ resistance [48].

Other sources of TMZ resistance include sub-populations of tumor cells with characteristics of cancer stem cells. Chen *et al.* have identified such a sub-population of endogenous glioma cells with characteristics of cancer stem cells that are responsible for transient bursts in proliferative cell populations and may account for GBM recurrence after standard therapy [49]. Others have speculated that abnormalities in the apoptotic pathway in cancer stem cell-like subpopulations lead to little response to therapy and later cancer recurrence [39]. Regardless, the behavior of such subpopulations in conditions of chemotherapeutic stress remains a complex phenomenon attributed to both intrinsic and extrinsic factors that contribute to the tumor microenvironment [40].

## 3. Radiation Therapy

While the superiority of radiation plus surgery over radiation alone has been known for decades, more recently it was shown radiation plus TMZ is superior to radiation therapy alone [33]. Stupp and colleagues demonstrated in their phase III trial that concomitant and adjuvant TMZ with radiotherapy in GBM produced a 2-year survival rate of 26.5%* versus* 10.4% survival in those who received radiotherapy alone; in so doing they established the current standard of care involving both TMZ and radiotherapy along with surgical resection [2,50]. Population-based studies have shown the most pronounced improvements in median OS after the introduction of TMZ and combined radiation therapy compared to prior periods of TMZ or radiation therapy alone [36].

Subsequent studies provided evidence that TMZ and radiotherapy act synergistically to promote tumor killing through TMZ-mediated radiation enhancement, specifically in those GBMs that have undetectable MGMT expression [25,51]. It is suggested that TMZ enhances dsDNA breaks in a radiation-induced pro-apoptotic environment. Those who have MGMT positive tumors can also benefit from the synergism of TMZ and radiotherapy when O^6^-benzylguanine is added concurrently, sensitizing resistant cells to TMZ [25]. Interestingly, Rivera *et al.* suggest that MGMT methylation may also predict response to radiotherapy, with methylated MGMT tumors having an overall improved response to radiotherapy in the absence of alkylating chemotherapy, while unmethylated MGMT tumors actually progress during radiation treatment (71%* vs.* 42% of individuals stable or response on post radiotherapy scan) [52].

While the standard dose of radiation therapy is 60 Gy, studies have shown that a maximum tolerated radiation dose of 75 Gy for locally aggressive tumors is safe and efficacious with concurrent TMZ therapy, resulting in no radiation necrosis and a median overall survival of 20.1 months in one study [53]. In the elderly, 50 Gy in 28 fractions has been shown to optimize improvement in survival without a concomitant decline in cognition or quality of life (median OS 29.1 weeks* vs.* 16.9 weeks with supportive care alone) [54]. Retrospective analyses have shown that concomitant, rather than adjuvant, TMZ with radiotherapy may be the superior regimen, resulting in a median overall survival of 17 months* vs.* 14.8 months in the EORTC study involving concomitant and adjuvant TMZ [2,55]. A recent phase III trial showed that in the elderly with newly diagnosed GBM, standard radiotherapy or hypofractionated radiotherapy did not confer increased survival over standard TMZ chemotherapy; as such, they recommended TMZ alone in the treatment of the elderly with GBM [56].

## 4. Current Immunotherapies

An intact immune system will imprint, or edit, the tumor with an immunologic signature ultimately impervious to immune activity [57]. Dunn and colleagues have described this process as the “Three Es” of immunoediting: Elimination, Equilibrium, and Escape [58]. Elimination involves immunosurveillance of the tumor, as the immune system destroys tumor cells it recognizes; equilibrium consists of the selection of tumor cells that cannot be destroyed as described above; escape reflects the process of aggressive resistant tumor growth. Facilitated by a tolerogenic tumor microenvironment that suppresses the cytotoxic activity of tumor infiltrating lymphocytes (TILs), poorly immunogenic tumors proliferate rapidly and often outpace even the destructive effects of chemotherapy and radiation. Targeted immunotherapies provide promise for combating tumor growth through immune activation of TILs and decreasing immune tolerance within the tumor microenvironment [59].

Immunotherapy centers on the principle that the host immune system may destroy tumor if immune effector function is appropriately augmented [60]. The immune system may eliminate tumor cells through recognition and processing of tumor antigens, tumor antigen presentation by antigen presenting cells via major histocompatibility complexes (MHC), or signaling through IFN-γ pathway constituents. In addition, the tumor may establish a locally immunosuppressive state by secreting cytokines such as VEGF and TGF-β, expressing galectin and the enzyme indeolamine 2,3-dioxygenase which depletes the microenvironment of amino acids necessary for lymphocyte anabolism [57]. Moreover, immunosuppressive cells such as Treg and myeloid derived suppressor cells (MDSCs) inhibit effector lymphocyte function. Tumor associated macrophages aid in tumor invasion and metastasis, and local regulatory B cells may produce pro-tumorigenic humoral responses. In addition to the aforementioned tumor strategies to evade the immune response, gliomas harbor unique immune evasion mechanisms. Such mechanisms include increased levels of immunosuppressive Tregs and decreased levels of effector CD8+ T cells in the glioma microenvironment, the lack or low level expression of co-stimulatory molecules in the brain, the expression of co-inhibitory molecules, such as B7-H1, on gliomas, and the presence of immunosuppressive glioma cancer stem cells that persist despite host anti-tumor immune responses [6,61,62,63,64,65,66].

There are three basic strategies underlying immunotherapy: immune modulating cytokine therapy, passive therapy and active immune therapy including cancer vaccines. These three strategies will be discussed as follows in the context of glioma and non-glioma tumor types, as many of these therapies have yet to be tested in glioma.

### 4.1. Cytokine Therapy

Cytokine therapy utilizes mediators of immune activation and proliferation to broadly induce an anti-tumor immune response. Cytokines that have been studied include gamma-chain cytokines, such as the interleukins IL-2, IL-7, IL-15, and IL-21 [67]; specifically, IL-2 has been approved for metastatic renal cell carcinoma for inducing high rates of regression [68]. IL-12 is a non-gamma chain cytokine that has also been relevant in cancer immunotherapy. Importantly, while cytokine therapy is effective at immune activation, the immune effects are non-specific and often lead to extensive systemic toxicities, limiting their use [67].

Cytokine IL-7 is involved in T-cell development and is expressed by stromal and parenchymal cells [69,70]. The effect of IL-7 in restoring the immune response and augmenting peripheral responses has been shown in pre-clinical models [71,72,73,74,75]. A phase I study demonstrated safety of a vaccine against renal cell carcinoma (RCC) containing tumor cells expressing RCC26/IL-7/CD80; while they did not observe increased Th1 specific immune responses, they did find higher levels of Th2 mediated IL-10 expression in patients who responded [76]. Moreover, another phase I study in refractory solid cancers demonstrated improved circulating CD3+, CD4+ and CD8+ T-lymphocyte profiles with only mild toxicities [77]. A number of phase I clinical trials in metastatic melanoma, RCC, and other solid tumors are currently underway, but the clinical effect of IL-7 in human glioma remains to be seen.

Unlike IL-7, IL-15 is not a soluble cytokine and is bound via its receptor to cells such as monocytes and DCs [78,79,80,81,82]. IL-15 is responsible for natural killer (NK) cell maturation and for the preservation of CD8+ memory T-cells, and this ability of inducing NK and CD8+ differentiation has garnered interest in its role in adoptive immunotherapy. Preclinical studies have shown increased potency of antigen-specific T-cells cultured with IL-15 in melanoma and plasmacytoma [83,84,85,86]. Phase I trials testing IL-15 are underway in a number of solid tumors, including melanoma, RCC, NSCLC, and squamous head and neck cancers.

IL-21 is produced only by activated CD4+ lymphocytes, but has stimulatory effects on CD4+, CD8+ and NK cells while inhibiting Treg function [87,88,89,90]. Preclinical studies with IL-21 show inhibition of tumor growth in melanoma and RCC and anti-tumor cytotoxic activity in murine genetically modified IL-21 secreting tumors [91,92,93,94,95]. A recent phase II study in metastatic melanoma treated with IL-21 demonstrated an overall response rate of 22.5%, median overall survival of 12.4 months and median progression free survival of 4.3 months [96]. Interestingly, given the dual effect of IL-21 in stimulating effector lymphocytes and inhibiting Th17 mediated responses, the combination of IL-21 and anti-PD-1 antibody is currently being studied in a phase I dose escalation study in advanced or metastatic solid cancers, and IL-21 combined with ipilimumab is being studied in a phase I trial in advanced melanoma (NCT01629758, NCT01489059). Preclinical studies of IL-21 are needed in GBM, but the clinical possibilities of combination immunotherapy are promising.

Produced by APCs, IL-12 is responsible for inducing Th1 immune responses and augmenting proliferation of NK and CD8+ T-cells, as well as engendering the production of IFN-γ, a marker of T-cell activation [97,98]. IL-12 therapy has resulted in increased tumor rejection and local inflammatory responses in mouse models of colon cancer, glioma, and hematologic malignancy [99,100,101,102]. Early clinical trials in ovarian cancer with intraperitoneal IL-12 plasmid vaccination have demonstrated safety and feasibility of delivery via a plasmid vector [103,104].

While cytokine therapy showed some potential in pre-clinical studies, initial clinical results in tumor regression have been disappointing. This may be due to the multiplicity of mechanisms of tumor immune evasion in the tumor microenvironment, such as immune checkpoint interactions, that continue to suppress immune activity despite the effects of cytokine therapy. Alternatively, local delivery of cytokine may be necessary to deliver a high enough intratumoral dose to be effective.

### 4.2. Passive Therapy: Immune Checkpoint Blockade and Adoptive Cell Therapy

Passive anticancer therapy utilizes antibodies to mediate cancer killing, either by targeting tumor antigen or preventing immune checkpoint-mediated inhibition. Antibody therapy that targets specific tumor antigen may promote tumor killing in one of three ways: 1. Direct receptor-mediated tumor cell apoptosis, 2. Immune-mediated tumor killing through macrophage phagocytosis, complement mediated destruction, or antigen presentation leading to T-cell activation, 3. Antagonism of vascular receptors and the destruction of vascular stromal cells, leading to cell death in the tumor parenchyma [105]. A number of antibodies have been FDA approved for the treatment of cancers, including antibodies conjugated to chemotherapeutic agents or radioisotopes as well as unconjugated antibodies that target pro-oncogenic factors, such as growth receptors [106,107]. One of the obstacles to successful antibody therapy, however, is immune escape of the tumor through the establishment of a locally immunosuppressive tumor microenvironment. A mechanism to overcome this challenge is the development of antibodies that target inhibitory immune pathways, termed immune checkpoints.

Antibody-mediated immune checkpoint blockade is one type of passive therapy that promotes immune effector function. Under normal circumstances, immune checkpoints are responsible for preventing autoimmunity during peripheral inflammatory responses. When bound to their respective ligands, immune checkpoint receptors may down-regulate or up-regulate immune activity through intracellular signal cascades. Malignant tumors may hijack this machinery by expressing ligands to inhibitory immune checkpoints, thereby depressing the activity of immune effector cells that may otherwise eradicate tumor. Examples of such inhibitory immune checkpoints include programmed death-1 (PD-1) and cytotoxic lymphocyte antigen-4 (CTLA-4) [108].

As immunotherapy gains traction as a putative treatment for glioblastoma, immune checkpoint inhibitors are gaining considerable attention as they show promise for the immunologic elimination of poorly immunogenic tumors. Parsa and colleagues have demonstrated a mechanism for GBM escape through loss of tumor suppressor gene PTEN, which results in overexpression of immunosuppressive B7 homolog 1 (B7-H1), also known as programmed death ligand-1 (PDL1) [6,109]. PDL1 is one component of an array of inhibitory immune checkpoint proteins that may be targeted to prevent immune dysregulation during tumor growth [108]. PDL1 expressed on host tissues binds to PD-1 receptor on activated lymphocytes in the periphery to prevent autoimmunity during inflammatory responses; engagement of PDL1 with PD-1 inhibits T-cell activation by inhibiting kinases via phosphatase SHP2 [110]. PD-1 is also highly expressed in regulatory T-cells (Treg) [108]. Through overexpression of PDL1 in GBM, tumor cells may exhaust activated T-cells, thereby precluding a robust immune response. Zeng and colleagues have reported that antibody blockade of PD-1 in mice with intracranial glioma combined with stereotactic radiosurgery (SRS) results in significantly improved survival [111]. While PD-1 alone provides modest survival benefit, the addition of SRS is key to eliciting a synergistic anti-tumor response in the mouse glioma model [111]. The underlying mechanism may be that SRS modifies the tumor microenvironment to produce inflammation, augmenting the anti-tumor immune response [112]. A phase I/II trial of anti-PD-1 therapy in relapsed GBM is currently underway (NCT01952769).

In addition to PD-1, immune checkpoint receptor CTLA-4 has begun to generate interest in its application as a part of combination immunotherapy. Located on T-cells, inhibitory CTLA-4 outcompetes stimulatory T-cell receptor CD28 for binding to their ligands CD80 and CD86, thus inhibiting T-cell effector function [110]. Unlike PD-1, CTLA-4 acts largely on naïve and resting T-cells. Ultimately, CTLA-4 downregulates helper T-cell activity and augments Treg activity [110]. Demaria and colleagues tested the effects of CTLA-4 blockade and SRS on breast cancer in mice; they reported that CTLA-4 blockade synergizes with SRS to significantly prolong survival, and that this survival benefit and prevention of distant metastases is largely mediated by CD8 T-cells [113]. Moreover, Wolchok *et al.* demonstrated that in a clinical trial of patients with melanoma, concurrent PD-1 and CTLA-4 blockade resulted in a 53% response rate with significant tumor regression and a manageable safety profile—this response rate far exceeded anything previously encountered with anti-PD-1 or anti-CTLA-4 monotherapy [114]. It is important to note, however, that the systemic autoimmune toxicities associated with checkpoint blockade are significant, including enterocolitis and hypophysitis [114,115]. Nevertheless, clinical results in immune checkpoint blockade in melanoma and other tumors are encouraging; these findings support further preclinical study of checkpoint inhibitors in glioblastoma as potential future therapeutics.

A second form of passive immunotherapy is adoptive T-cell therapy, in which tumor-specific T-cells are activated *ex vivo* and transferred to the patient [116,117,118]. As the immune cells are activated before coming into contact with tumor, it is less likely that they will succumb to the inhibitory effects of the tumor microenvironment. Transfer of tumor reactive T-cells to patients with melanoma have led to objective responses of 34%–50% [119,120]. Objective response rates up to 30% have been observed in patients with melanoma receiving adoptive T-cell transfers expressing genetically altered T-cell receptors (TCRs) that have higher affinities for MHC-antigen complexes [121]. A number of phase I and II clinical studies have been completed or are underway testing adoptive T-cell therapies in GBM (NCT00331526, NCT01082926, NCT00693095, NCT01588769, NCT01454596, NCT00730613, NCT01109095).

### 4.3. Active Therapy: Vaccines

Vaccine therapy aims to break immune tolerance of tumors by exposing antigen presenting cells (APCs) to immunogenic tumor peptides in the hopes of activating a broad lymphocytic immune response involving both CD4+ and CD8+ T-cells. Recent advances in cancer vaccine therapy have been achieved in hormone-resistant prostate cancer and advanced melanoma [122,123].

Cancer vaccines have emerged as a therapy that offers the following unique advantages: vaccine-stimulated immune cells (1) have high specificity for tumor, (2) are able to kill target cells in all stages of the cell cycle, unlike checkpoint inhibitors, (3) can target tumor cells with intrinsic or acquired drug resistance, a major obstacle for current chemotherapeutic regimen [39,124], (4) can confer immunologic memory and durable long-term protection, and (5) can potentially be used in the future for preventative immunization in high-risk populations [21,125]. Immunotherapy can also be dynamic in that the immune response can adapt to changes in antigen expression of the tumor, a phenomenon known as epitope spreading (REF).

#### 4.3.1. Dendritic Cell Vaccine

Dendritic cells are the most potent APCs of the immune system with the ability to induce lasting immunity in naive individuals [126,127,128]. DC vaccines are capable of inducing systemic immunity through a number of different *ex vivo* and *in vivo* approaches, including DCs loaded with tumor lysate, MHC I derived peptides, or tumor apoptotic bodies as well as direct intratumoral injection [129,130,131,132]. Because DCs have the capability of priming the immune system through tumor antigen presentation to T-cells, DC vaccines are capable of orchestrating a robust cell-mediated immune response against infiltrating tumor cells. By exposing DCs to tumor antigen either *ex vivo* or *in vivo*, DC vaccines facilitate tumor destruction via the body’s own adaptive immune response.

In an early study, Mayordomo and colleagues demonstrated that DCs pulsed with MHC I restricted tumor-associated peptides initiated a T-cell immune response that was protective after subsequent tumor challenge and induced 80% regression in established C3 sarcoma and 3LL lung carcinoma mouse models [133]. Subsequently, Liau *et al.* produced prolonged survival in a rat glioma model treated with DCs pulsed with various tumor antigens [134]. It was quickly recognized that the primary limitation of DC vaccines is the necessity of direct physical interaction of DCs and tumor antigens, preferably *ex vivo* as DCs are largely incapable of processing tumor antigens in the immunosuppressive tumor microenvironment [127,135].

Nevertheless, a series of phase I and II clinical trials proved promising for DC vaccination as an effective immunotherapy for GBM (Table 2). Phase I trials demonstrated enhanced antigen-specific cytoxotic T-cell responses in both peptide- and tumor lysate-pulsed vaccine formats [136,137,138,139]. Specifically, Yu *et al.* found that treatment of malignant glioma with tumor lysate-pulsed DC vaccine produced a median survival of 133 weeks, while Liau and colleagues showed that a robust intratumoral cytotoxic T-cell response was elicited after treatment with peptide-pulsed DC vaccination that positively correlated with survival (*p* = 0.047) [138]. Of note, Liau* et al.* also found that TGF-β2 expression negatively correlated with the degree of intratumoral T-cell infiltrate as well as survival and clinical response, possibly due to its immunosuppressive effects on T-lymphocytes [138]. Moreover, Yamanaka and colleagues showed an enhanced overall survival of 480 days in those with grade 4 glioma treated with tumor lysate-pulsed DC vaccine compared to 400 days in the control group, with the most improved survival in those receiving both intradermal and intratumoral vaccination; such differences in survival could be attributed to activation of both central and peripheral immune responses [139]. An important step in the development of DC vaccine therapy for GBM occurred in a study by Wheeler *et al.* which demonstrated correlated immune and clinical responses in those with grade 4 glioma. They found that 53% of GBM patients treated with vaccine exhibited ≥1.5 fold cytokine responses and statistically significant time to progression and time to survival as well as a 2-year survival rate of 41% (*p* = 0.03) [140].

Another potential limitation of DC vaccines was the assumption that multi-antigenic vaccines would result in catastrophic autoimmunity, and that the safest vaccines would utilize single tumor antigens. However, a number of studies have incorporated multi-antigenic DC vaccines without the predicted autoimmunity and have demonstrated clinical anti-tumor effects [136,137,141,142,143]. One of these studies is a phase I trial that showed that ICT-107, an autologous multi-antigenic DC vaccine utilizing six GBM peptides, produced a median overall survival of 38.4 months and a progression free survival of 16.9 months; a phase II trial studying ICT-107 is currently underway [143]. In all, DC vaccines show promising immune and clinical responses in early clinical trials, but phase III trials are needed to confirm the clinical efficacy of such immunotherapy. Moreover, whether the evolving tumor microenvironment overcomes the immunity induced by DC vaccination remains to be seen [58,128,144,145].

**Table 2:** Phase I and II clinical trials measuring cancer vaccine safety and efficacy in Glioblastoma.

Vaccination Type	Major Findings	Reference
Autologous glioma cell peptide-pulsed DC vaccine	Systemic cytotoxicity in 4/7 Intratumoral CD8+ T-cell infiltration in 2/4	^1^ Yu, *et al.* 2001 [136]
Autologous tumor lysate-pulsed DC vaccine	Systemic cytotoxicity in 6/10 Intratumoral CD8+ T-cell infiltrate in 3/6	^1^ Yu, *et al.* 2004 [137]
Acid-eluted autologous glioma peptide-pulsed DC vaccine	Systemic anti-tumor cytotoxic T-cell response in 6/12 Intratumoral CD8+ T-cell infiltrate in 4/8	^1^ Liau, *et al.* 2005 [138]
Autologous tumor lysate-pulsed DC vaccine	Median OS 480 days (*p* = 0.01)	^2^ Yamanaka, *et al.* 2005 [139]
Autologous tumor lysate-pulsed DC vaccine	TTS 642 ± 61 days (*p* = 0.041)	^3^ Wheeler, *et al.* 2008 [140]
EGFRvIII peptide DC vaccine	Median OS 22.8 months	^1^ Sampson, *et al.* 2009 [141]
EGFRvIII peptide vaccine	Median OS 26 months	^2^ Sampson, *et al.* 2010 [17]
EGFRvIII peptide vaccine	Median OS 23.6 months	^2^ Sampson, *et al.* 2011 [142]
EGFR, EZH2, Lck, MRP3, PAP, PSA, PSMA, PTH-rP, SART peptide vaccine	Median OS 10.6 months	^1^ Terasaki, *et al.* 2011 [143]
AIM-2, MAGE1, TRP-2, gp100, HER2/neu, IL-13Rα2	Median OS 38.4 months	^1^ Phuphanich, *et al.* 2012 [146]

Selected major studies included that report clinical efficacy of cancer vaccines for GBM. Major findings refers to significant clinical responses reported in each study. ^1^ Phase I trial. ^2^ Phase I/II trial. ^3^ Phase II trial. DC = Dendritic Cell. OS = Overall Survival. TTS = Time to Survival.

#### 4.3.2. EGFRvIII Vaccine

A promising antigen for peptide vaccination is epidermal growth factor receptor (EGFR), a transmembrane tyrosine kinase receptor that is rearranged and overexpressed in nearly half of malignant gliomas, resulting in structural rearrangements and enhanced oncogenicity [147,148,149,150]. The most commonly observed mutation of EGFR in human neoplasms is variant III (EGFRvIII), contributing to increased tumorogenicity in primary and secondary brain tumors [150,151,152,153,154,155,156]. Wong and colleagues were the first to describe increased expression of the *EGFR* gene in gliomas with amplified *EGFR* as well as characterize the structural rearrangements of EGFR in malignant gliomas [147,148]. Heimberger *et al.* found that the expression of mutant EGFRvIII in GBM is an independent negative prognostic marker, and tumor specific antibodies to EGFRvIII have been found in breast cancer, head and neck cancers, and other tumors, with the absence of EGFRvIII in normal tissues [149,151,152,153,154]. Moreover, a number of studies have established EGFRvIII as an oncogene, enhancing tumorigenicity through increased cell motility, migration, and consequent invasiveness [155,156,157]. Increased expression of EGFRvIII promotes cell proliferation and inhibits apoptosis, which has been shown to confer chemo- and radio-resistance in a number of different tumors [158,159,160].

Given that EGFRvIII is expressed exclusively in malignant tissue and is responsible for enhanced tumorigenicity, it is a logical target for immunotherapy. Preclinical data in mice showed that peptide vaccination against the mutated segment of EGFRvIII (PEP-3) prevented melanoma tumor development in 70% and that sera from immunized mice was protective in 31% of non-immunized mice implanted with tumor. Moreover, mice treated with DC vaccine pulsed with EGFRvIII produced an antigen-specific immune response, leading to long-term anti-tumor immunity [149,161]. Clinical trials have shown EGFRvIII-specific humoral responses are detectable in GBM patients vaccinated with PEP-3 and that anti-EGFVRvIII DC vaccines are safe and efficacious immunotherapeutic alternatives or adjuncts to standard therapy [141,162] (Table 2). A recent phase II clinical trial demonstrated a significant increase in median overall survival (26 months, *p* = 0.0013) after vaccination against EGFRvIII in GBM compared to non-vaccinated historical controls [17]. However, the same trial also showed immunologic escape as 82% of individuals had lost tumoral EGFRvIII expression at GBM recurrence. Currently, a phase III clinical trial (ACT IV) is underway studying the effects of CDX-110 (PEPvIII-KLH vaccine) in newly diagnosed, EGFRvIII positive GBM. This is a randomized, double-blind trial in which patients are randomized to either the CDX-110 treatment arm or the keyhole limpet hemocyanin (KLH) control arm; both arms will receive standard TMZ therapy and will be followed until death. The ReAct trial is a phase II study currently underway examining the effects of CDX-110 in recurrent GBM refractory to bevacizumab therapy. Patients with recurrent GBM who have never been treated with bevacizumab are randomized to receive either CDX-110 or the control KLH, while patients who are refractory to bevacizumab will receive CDX-110 and bevacizumab. Patients are followed for survival.

Preclinical and early clinical data regarding the EGFRvIII vaccine is encouraging. In addition to EGFR, others have raised the possibility of personalized peptide vaccines or even individualized whole tumor vaccines as more targeted alternatives for immunotherapy [143,163,164].

#### 4.3.3. Heat Shock Proteins

Heat shock proteins (HSPs) are chaperones responsible for facilitating protein folding and are overexpressed during cellular environmental stress, such as hyperthermia, UV irradiation, or oxidative stress [165,166,167]. Moreover, HSPs are responsible for mediating apoptotic pathways and capable of stimulating antigen-specific cytotoxic T-cell responses through the action of APCs [168,169,170,171,172]. Given that they are highly immunogenic and tumor-specific, HSPs are attractive antigens for vaccine therapy [173]. Heat shock protein-96 peptide complex (HSPPC-96) has been developed as an autologous tumor antigen peptide vaccination for GBM, and is currently being tested in phase II clinical trials for those with recurrent and newly diagnosed GBM [174,175]. Preliminary trial results demonstrate a median survival of those treated with HSPPC-96 of 44 weeks compared to the historical median survival of 26 weeks [176]. Such results are encouraging for the future use of HSPs in potent anti-cancer vaccines alongside standard therapy.

## 5. Combining Vaccine Immunotherapy with Standard of Care

The persistence of GBM refractory to chemotherapy and radiation has provided the impetus for exploring other therapeutic avenues, including new antigens for vaccines and multi-modality therapy that combines immunotherapy with the current standard of care. While cytotoxic therapy may decrease tumor volume, survival remains poor as a result of the resilient GBM tumor microenvironment. As described previously, the tumor cell expression of molecules that inhibit immune effector function preclude tumor elimination. Immunotherapy provides promise to overcome such immunologic obstacles [57,59]. The following sections will explore novel antigens for peptide vaccines as well as the promise of combination immunotherapy alongside standard of care.

### 5.1. Peptide Vaccines

#### 5.1.1. Novel Peptide Vaccines

Peptide vaccines consist of small peptide antigens that are expressed by the target tumor cells and are injected subcutaneously. Capable of creating complexes with HLA class I antigen, these peptides are digested by host APCs and subsequently presented to cytotoxic T-lymphocytes (CTLs) in the lymph nodes. Once sensitized and after clonal expansion, the CTLs disseminate systemically and destroy tumor upon recognition of the peptide-HLA complex correlate on tumor cells. In this manner, peptide vaccines prime the adaptive immune system to eliminate tumor and promote tumor regression. Use of normal gene products as vaccine targets carries an inherent risk of autoimmune toxicity. However, several antigens that are expressed in immunoprivileged or tissue-specific sites of the body have been characterized as potentially effective antitumor targets [177,178]. These new antigens could be targets for future therapies (Table 3).

**Table 3:** Selected tumor antigens targeted in glioma vaccination studies.

Antigen	Significance	Examples of Use in Glioma Vaccines
TRP-2 (tyrosinase-related protein-2)	Human melanoma-derived tissue differentiation antigen present in 50% of GBM-derived cell lines [179]. Overexpression associated with drug and radiation resistance.	Wheeler, *et al.* [18] Liu,* et al.* [180] Liu*, et al.* [181]
HER2	Well-defined oncogenic protein and tumor antigen present with high frequency in breast, ovarian, renal cell carcinoma, and colon cancers. Documented expression in human GBM cells and recognized by cytotoxic T cells [182]	Phuphanich, *et al.* [146] Yu, *et al.* [137]
gp100 (human melanoma-associated antigen)	Highly immunogenic antigen in melanoma found to be expressed in GL26 and GL261 glioma cell lines [183] and recognized by CTLs [184]	Phuphanich, *et al.* [146] Okada, *et al.* [185] Yu, *et al.* [137]
MAGE-1 (melanoma-associated antigen)	Immune-stimulating testis tumor antigen group with four-fold higher expression in GBM than normal astrocytes. Overexpression associated with chemotherapy resistance in ovarian [186], gastric [187] and medulloblastoma [188] cancer cell lines.	Wheeler, *et al.* [18] Reddy, *et al.* [189] Phuphanich, *et al.* [146] Yu, *et al* [137]
EGFRvIII	Mutant transmembrane tyrosine kinase receptor overexpressed in nearly half of all malignant gliomas. Associated with resistance to chemotherapy and radiation therapy [149,151,152,153,154,155,156,157,158,159,160]	Heimberger, *et al.* [149] Heimberger, *et al.* [161] Sampson, *et al.* [17]
IL13Rα2	Cell surface receptor overexpressed by a subset of high grade gliomas [184,190]. May be overexpressed by treatment refractory glioma stem cells, rendering them susceptible to targeted CTLs [191]	Phuphanich, *et al.* [146] Pollack, *et al.* [192]
AIM-2 (Antigen isolated from Immunoselected Melanoma-2)	Tumor antigen expressed in melanoma, as well as neuroectodermal, breast, ovarian and colon cancer cells [193]. Overexpressed in human glioma cells and recognized by CTLs [182].	Phuphanich, *et al.* [146]

An overview of major antigenic peptides used in glioma antitumor vaccines.

#### 5.1.2. TRP-2

Tyrosine-related protein-2 (TRP-2) is a normal tissue differentiation antigen from human melanoma that is recognized by T cells [194] (Table 3). A study by Liu *et al.* demonstrated TRP-2 was present in over 50% of GBM-derived cell lines. Furthermore, the antigen was not only expressed on the glioma cell surface, but also processed by its major histocompatibility complexes (MHCs) and presented to cytotoxic T cells [180]. Wheeler *et al.* observed that patients who received chemotherapy after active TRP-2-bearing dendritic cell vaccination experienced slower progression and longer survival than patients who received either chemotherapy or vaccination alone [18]. A subsequent human study by the same group established an association between the presence of TRP-2 specific cytotoxic T cell (CTL) activity post-vaccination and significantly reduced TRP-2 expression in resected glioma cells. Furthermore, these cells demonstrated higher sensitivity to carboplatin and TMZ [181]. Overexpression of TRP-2 has been associated with drug and radiation resistance through activation of the extracellular signal regulated kinase (ERK)/mitogen-activated protein kinase (MAPK) pathway [194,195]. Thus, the study by Liu *et al.* provides compelling evidence that vaccine-mediated depletion of TRP-2 expressing glioma cells could undermine tumor resistance to the current standard of care. Moreover, a recent phase I trial of a multi-epitope DC vaccine containing TRP-2 for newly diagnosed GBM has demonstrated prolonged overall survival and progression free survival [146].

#### 5.1.3. MAGE-1

The melanoma-associated antigen (MAGE) family of peptides belongs to the cancer/testis tumor antigen group, which are normally expressed in immunoprivileged testicular tissue. Originally identified as an immune stimulating antigen found in 50% of melanomas, MAGE-1 has been shown to have four-fold higher expression in glioblastoma than in normal astrocytes [196,197]. MAGE-1 was another target of dendritic cell vaccines that was found by Wheeler *et al.* to sensitize tumors to subsequent chemotherapy [18]. At present, there are no studies that implicate MAGE-1 in glioma drug resistance. However, MAGE and GAGE gene overexpression has been associated with resistance to paclitaxel and doxorubicin in human ovarian cancer cell lines [187]; docetaxel and paclitaxel in advanced and recurrent gastric cancer [188]; and cisplatin and etoposide in medulloblastoma cell lines and specimens [189]. In contrast, a study by Reddy *et al.* demonstrated that overexpression of Dixin-1, a MAGE family antigen, inhibited proliferation, invasion, and tumorigenicity in highly-chemo-resistant glioma stem cells [186]. Thus, the role of MAGE-1 vaccines in drug-resistant tumors is unclear and requires further investigation.

### 5.2. Standard of Care and Immunotherapy: Opportunities for Synergism

#### 5.2.1. Vaccines and Chemotherapy

Chemotherapy and immunotherapy have often been regarded as independent or antagonistic treatment modalities. This assumption is based on two considerations. First, cytotoxic chemotherapy is associated with severe lymphopenia, due to non-specific cell death of proliferating cells [183,198,199]. Second, chemotherapy-induced cell death was presumed to occur through a non-inflammatory apoptotic process or by induction of immune tolerance [183,198,199]. In the first case, lymphopenia could theoretically curtail immunotherapy’s effectiveness by depleting the peripheral pool of immune cells available for mounting an antitumor response. In the second case, chemotherapy’s effects would be either a passive lack of immune activation and proliferation or an active suppression of antigen presentation by tumor infiltrating T cells [198,200]. However, new evidence has called these assumptions into question.

In a 2008 case study by Heimberger *et al.* successful immune activation was demonstrated after concomitant treatment with TMZ and EGFRvIII vaccine [201]. In addition, notable was the lack of CD4 and CD8 T cell count decline after sequential courses of chemoradiation and vaccination. The authors suggest that chemotherapy is not necessarily counterproductive to immunotherapy as long as it is administered outside of the effector window, during which vaccine-induced CTLs are most responsive and active against the tumor. Outside this effector stage, the lymphodepleting effects of TMZ may actually be desirable, as it can reduce immunosuppressive lymphocytes such as Tregs, and thereby improve the local immune profile [201,202]. A subsequent study by Banissi *et al.* found that low-dose metronomic TMZ administration in a rat glioma model resulted in decreased circulating Tregs and reduced tumor progression, though the latter did not reach statistical significance [203]. These studies serve to emphasize the delicate immune balance that can ultimately determine the success of a treatment protocol. In practice, clinicians may either administer vaccines concurrently with chemoradiation, or use an interdigitated alternating regimen as a strategy to minimize CTL depletion and maximize Treg inhibition.

In addition to harnessing chemotherapy’s immune-activating effects, immunotherapy may also be used as chemo-sensitizing agents that render drug-resistant tumors more amenable to standard therapeutic options [21]. In 1982, van Pel *et al.* demonstrated that tumors with apparently no transplantation immunogenicity still displayed enough foreign antigens to elicit a syngeneic rejection response [204]. Subsequent studies of brain tumors have focused on identifying immunologically susceptible GBM antigen profiles that may be exploited as targets for vaccines.

Ever since the feasibility and safety of synthetic peptide vaccines were demonstrated in clinical trials [205,206], concerted efforts have been made to identify MHC-restricted GBM antigens that can be delivered in the form of isolated peptides or peptide-bearing APCs. These antigens are often characterized as a product of either normal gene overexpression (*i.e.*, TRP-2, MAGE-1) or a mutated somatic gene (EGFRvIII) [177,178]. Furthermore, certain antigens have been associated with a drug or radiation resistant phenotype, thereby providing a target to overcome these treatment barriers. Antigens that are suspected to play a role in synergy between drug and vaccines therapies, including TRP-2, MAGE-1, and EGFRvIII have been discussed previously (refer to Section 5.1: Peptide Vaccines).

#### 5.2.2. Vaccines and Radiation Therapy

RT is commonly considered to be an immunosuppressive agent that non-selectively targets cells undergoing rapid division [207]. T lymphocytes, in particular, have been shown to be extremely sensitive to ionizing radiation [208]. Thus, it is natural to assume that radiotherapy is counterproductive to immunotherapy, as it systematically eliminates key mediators of the antitumor response. In a study by Grossman* et al.*, patients treated with radiation and TMZ for high grade gliomas experienced a CD4 count nadir at 2 months after treatment initiation. Treatment-induced CD4 count suppression was found to be not only long-lasting, but also associated with earlier death secondary to tumor progression [209]. Similar drops in CD4 counts were seen after treatment with steroids and radiotherapy only [210], further associating radiation with local and systemic immune compromise.

However, in addition to its cytocidal effects, RT has been shown to induce significant cellular and stromal changes by triggering “danger” signals that activate the innate and acquired immune system [211,212,213]. Irradiated apoptotic tumor cells are phagocytosed by potent APCs such as DC’s, which process and present tumor-specific antigens on MCH class 1 complexes, thereby activating CD8+ CTLs through the endogenous pathway [214,215,216]. Immune activation secondary to apoptotic cell death suggests a synergistic role for radiation therapy and immunotherapy. When used in conjunction with anti-CTLA-4 antibodies, RT has been associated with an immune-mediated inhibition of tumor cells outside the irradiated field—also known as the abscopal effect [217,218]. A recent study by Zeng *et al.* further showed that combined use of anti-PD1 antibodies and localized RT increased expression of MHC class I, CXCL16, and ICAM molecules * in vitro*, and resulted in a survival benefit in orthotopic GBM mouse models [111]. These findings strongly suggest a proinflammatory effect of radiotherapy that may synergize with therapeutic immune modulators.

MHC downregulation is a well-described phenomenon in invading glioma cells [219,220]. RT increases expression of MHC molecules, thereby counteracting a principal strategy for immune evasion by GBM. Increased antigen presentation has an added benefit of providing a natural target for vaccine-induced antitumor immunity. Using GL261 glioma mouse models, Newcomb *et al.* observed *in vivo* and upregulation of β2-microglobulin light chain subunit of the MHCI complex on the glioma cells, with a concomitant increase in CTL and helper T cell infiltration after whole body radiation therapy (WBRT). Furthermore, administration of both WBRT and allogeneic GL261 vaccination resulted in a survival advantage compared to WBRT alone, with superior long-term survival from 40% to 80% after combination therapy and 0% to 10% with monotherapy [19].

The synergistic effects of radiation-induced tumor necrosis and DC therapy have also been reported. Exposure to necrotic cells has been shown to induce DC maturation and enhanced host antitumor response secondary to cross-priming with effector lymphocytes [132,215]. The survival benefits of combined RT and DC vaccination have been described in several tumor models including melanoma [221], prostate cancer [222], liver metastasis [49], and mammary tumors [223]. In a study by Kikuchi *et al.* intratumoral injection of either immature DCs or irradiated necrotic glioma cells (IR-GCs) alone had no effect on survival in glioma mouse models. However, combined administration of DCs and IR-GCs resulted in synergistic antitumor effects and prolonged survival [224].

Several studies have delved into the synergistic effects of EGFR vaccines and RT. As stated previously, EGFRvIII overexpression is a poor prognostic indicator. Radiation exposure has been shown to result in robust stimulation of the EGFRvIII mutant receptor, leading to increased tumor survival and expansion [160,225]. The mechanism for enhanced clonogenic survival after RT may be EGFR-mediated activation of the phosphatidylinositol 3-kinase (PI3K)/Akt-1 pathway, leading to accelerated repair of double-stranded DNA breaks, thereby abrogating RT cytotoxicity [225]. Gefitinib (an EGFR inhibitor) and LY294002 (PI3K inhibitors) have been shown to attenuate repair of double-stranded breaks [226]. As of yet, there are no studies that specifically examine the synergistic effects of the EGFRvIII vaccination and RT. However, the existing evidence suggests that there may be a role for peptide vaccines in the radiosensitization of malignant gliomas.

## 6. Conclusions and Future Directions

Despite aggressive treatment with the current standard of care (maximal surgical resection with adjuvant radiotherapy and TMZ), 5-year survival for GBM remains at a dismal <2% [6]. Immunotherapy has potential as a future adjunct to standard of care; cancer vaccines such as the dendritic cell (DC) and epidermal growth factor receptor (EGFR) vaccines have shown encouraging results in phase I and II clinical trials and raise the possibility of personalized peptide vaccines or even individualized whole tumor vaccines as more targeted alternatives for immunotherapy. Synergy between cancer vaccines and conventional chemotherapy and radiation therapy has also demonstrated a potential role for immunotherapy in multi-modal treatment paradigms. Vaccines against tumor-associated antigens such as TRP-2 and MAGE-1 and ongogenic mutations such as EGFRvIII may facilitate host antitumor immunity and prolonged survival. Additional studies advocate a role for peptide vaccines in the radiosensitization of malignant gliomas. Further research on the interactions between chemotherapy, radiation, and immunotherapy is needed to incorporate cancer vaccines into the current standard of care, and to maximize the antitumor potential of each treatment modality.
